# Bridging borders: adapting the Orange County methicillin-resistant *Staphylococcus aureus* decolonization protocol for an infirmary unit in Hong Kong

**DOI:** 10.1017/ice.2025.10388

**Published:** 2026-04

**Authors:** Shuk-Ching Wong, Germaine Kit-Ming Lam, Raveena D. Singh, Edwin Kwan-Yeung Chiu, Kelvin Hei-Yeung Chiu, Pui-Hing Chau, Jonathan Daniel Ip, Bingpeng Yan, Simon Yung-Chun So, Wai-On Tam, Patrick Ka-Chun Chiu, Kong-Hung Sze, Edmond Siu-Keung Ma, Kwok-Yung Yuen, Susan S. Huang, Vincent Chi-Chung Cheng

**Affiliations:** 1 Infection Control Team, Queen Mary Hospital, Hong Kong West Cluster, Hong Kong Special Administrative Region, Hong Kong, China; 2 School of Nursing, Li Ka Shing Faculty of Medicine, The University of Hong Kong, Pokfulam, Hong Kong Special Administrative Region, Hong Kong, China; 3 Department of Microbiology, School of Clinical Medicine, Li Ka Shing Faculty of Medicine, The University of Hong Kong, Pokfulam, Hong Kong Special Administrative Region, Hong Kong, China; 4 Infection Control Team, Grantham Hospital, Hong Kong West Cluster, Hong Kong Special Administrative Region, Hong Kong, China; 5 Division of Infectious Diseases, University of California, Irvine School of Medicine, Irvine, CA, USA; 6 Department of Microbiology, Queen Mary Hospital, Hong Kong Special Administrative Region, Hong Kong, China; 7 Division of Geriatric Medicine, Grantham Hospital, Hong Kong Special Administrative Region, Hong Kong, China; 8 Centre for Health Protection, Department of Health, Hong Kong Special Administrative Region, Hong Kong, China

## Abstract

**Background:**

This study aims to evaluate the effectiveness of an adapted methicillin-resistant *Staphylococcus aureus* (MRSA) decolonization program in an infirmary unit in Hong Kong that was inspired by successful interventions implemented in Orange County, California.

**Methods:**

Nasal, skin, and rectal swabs were collected to assess MRSA colonization. Decolonization involved applying 10% povidone-iodine ointment to the anterior nares twice daily for five days every other week, along with twice weekly chlorhexidine gluconate (CHG) bathing for six months. Compliance with the application of povidone-iodine and CHG bathing techniques was monitored by measuring their respective levels in the anterior nares and on the skin. Air and environmental samples were collected and analyzed over time using linear regression.

**Results:**

Among 60 patients in the infirmary unit (78% baseline MRSA carriers), overall MRSA colonization declined during the program, driven by significant reductions in skin colonization (65% to 29%, *P* < .001). Environmental contamination on high-touch patient-care equipment (bathing trolleys and slings) also significantly decreased over time (*P* < .001). These reductions coincided with the high-quality implementation of decolonization, evidenced by stable iodophor detection in nares during application weeks and sustained chlorhexidine levels on the skin, detectable 24 hours after bathing. In contrast, MRSA detection in air samples showed no significant change (*P* = .096), possibly due to dispersal by persistent carriers during care activities even as skin and environmental contamination declined.

**Conclusions:**

The adapted MRSA decolonization program was effective, significantly reducing overall MRSA colonization, especially at skin sites, while achieving high compliance with the protocol.

## Introduction

Since the emergence of methicillin-resistant *Staphylococcus aureus* (MRSA) in the 1960s, it has spread globally and become endemic, posing a significant challenge to healthcare settings worldwide.^1,2^ MRSA is a major cause of healthcare-associated infections, contributing to substantial morbidity and mortality.^3^ To mitigate the risk of nosocomial MRSA transmission, numerous international guidelines have evolved to recommend comprehensive infection prevention and control measures, including active surveillance cultures, hand hygiene, contact precautions, cohort nursing or single-room isolation, environmental disinfection, antimicrobial stewardship, decolonization, and thorough patient education.^4–7^

In resource-limited settings such as Hong Kong, controlling nosocomial MRSA transmission remains a priority in the hospitals.^8,9^ The One Health approach to antimicrobial resistance control in Hong Kong emphasizes the importance of addressing MRSA prevalence not only in hospitals but also in residential care homes for the elderly (RCHEs).^10^ The Centre for Health Protection of the Department of Health in Hong Kong publicly reports MRSA data collected from public hospitals.^11^ Our previous local study demonstrated that MRSA transmission dynamics in RCHEs are approximately three times higher than those observed in hospital settings.^12^ Moreover, the prevalence of MRSA colonization in RCHEs has progressively escalated from less than 3% to 48% over the past two decades.^13–18^

Given these findings, we hypothesize that the MRSA burden in infirmary units is comparable to that in RCHEs or nursing homes. Building on the successful MRSA decolonization program implemented in nursing homes in Orange County, Los Angeles, USA,^19,20^ we adapted and modified the protocol to implement an MRSA decolonization strategy in an infirmary unit in Hong Kong. We describe the adaptation process and evaluate the effectiveness of this intervention in our local context.

## Material and methods

### Setting

**Figure 1.**
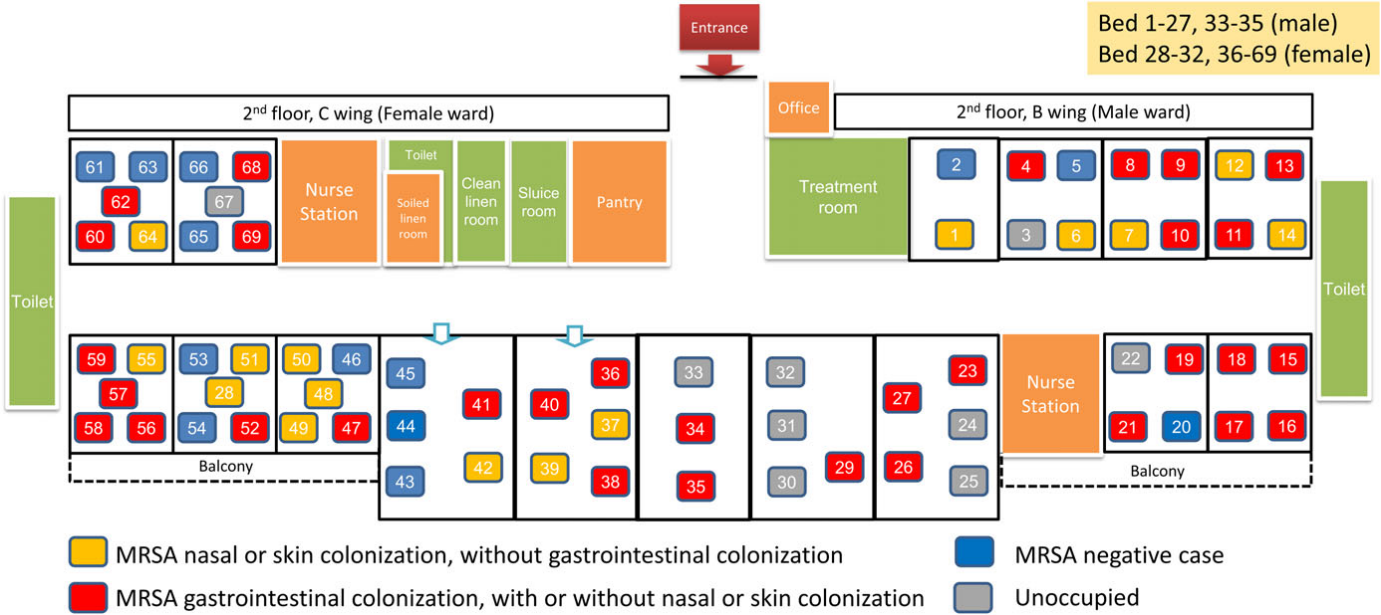
Floor plan of the Infirmary Unit, Grantham Hospital and distribution of MRSA colonization cases before the commencement of MRSA decolonization. *Note*: The B wing of the 2nd floor (Ward 2B) is designated for male patients, while the C wing of the 2nd floor (Ward 2C) is designated for female patients. In the infirmary unit, bed numbers may not be assigned consecutively due to geographic conditions. A bed number is assigned to each patient upon admission. If a patient needs to transfer between a side room and an open cubicle, or vice versa, due to medical requirements, the original bed number will be reassigned in the new area. For example, bed 28 is located in the cubicle designated for beds 51–54. Air samples were collected using settle plate methods, with ChromID MRSA agar (bioMerieux, France) plates placed at 30 designated positions, on top of the window air conditioner within the cubicle or side room, unless specified otherwise, once weekly during the MRSA decolonization period. Position 1 refers to the side room for beds 1–2; Position 2 refers to the cubicle for beds 3–6; Position 3 refers to the cubicle for beds 7–10; Position 4 refers to the cubicle for beds 11–14; Position 5 refers to the patient toilet in Ward 2B; Position 6 refers to the cubicle for beds 15–18 (on top of exhaust fan A); Position 7 refers to the cubicle for beds 15–18 (on top of exhaust fan B); Position 8 refers to the cubicle for beds 19–22 (on top of exhaust fan C); Position 9 refers to the cubicle for beds 19–22 (on top of exhaust fan D); Position 10 refers to the nursing station in Ward 2B; Position 11 refers to the nursing station in Ward 2B (on top of window air conditioner); Position 12 refers to the side room for beds 23–27; Position 13 refers to the side room for beds 29–32; Position 14 refers to the side room for beds 33–35; Position 15 refers to the side room for beds 36–40; Position 16 refers to the side room for beds 41–45; Position 17 refers to the cubicle for beds 46–50; Position 18 refers to the cubicle for beds 51–54 with insertion of bed 28; Position 19 refers to the cubicle for beds 55–59; Position 20 refers to the patient toilet in Ward 2C; Position 21 refers to the cubicle for beds 60–64; Position 22 refers to the cubicle for beds 65–69; Position 23 refers to the nursing station in Ward 2C; Position 24 refers to the nursing station in Ward 2C (on top of window air conditioner); Position 25 refers to the soiled linen room; Position 26 refers to the clean linen room; Position 27 refers to the sluice room; Position 28 refers to the treatment room; Position 29 refers to the patient toilet in Ward 2B (during bathing); Position 30 refers to the patient toilet in Ward 2C (during bathing).

This study was conducted in the infirmary unit at Grantham Hospital (GH), a 400-bed extended care facility within the Hong Kong West Cluster, governed by the Hospital Authority.^21^ The unit serves older adults or disabled individuals requiring long-term medical and nursing care, akin to skilled nursing facilities in the USA, but with services that exceed those typically offered by RCHEs.^22^ The infirmary unit includes two wards: Ward 2B for male patients and Ward 2C for female patients, along with a corridor of six single rooms adaptable for either gender as previously described^23^ (Figure 1). Infection control services are provided by GH’s Infection Control Team, which includes an infection control officer and infection control nurse (ICN), who collaborate with clinical staff on hand hygiene, vaccination, and outbreak prevention.

### Epidemiology of MRSA colonization among patients in the infirmary unit

Prior to the MRSA decolonization program, nasal swabs, skin swabs (axilla and groin), and rectal swabs were collected to assess MRSA colonization among patients in the infirmary unit. Newly admitted patients were also screened. Samples were collected by a trained nurse, the ICN at GH, and sent to the clinical microbiology laboratory for analysis. Demographic data, underlying chronic diseases, the presence of indwelling devices (such as nasogastric tubes, tracheostomies, and urinary catheters), and duration of hospital stay were also recorded for analysis.

### MRSA decolonization among patients in the infirmary unit

As part of infection control and quality improvement initiatives, MRSA decolonization was implemented on January 1, 2025, for all patients in the infirmary unit, who provided verbal consent, regardless of their MRSA colonization status. The regimen was adapted from studies conducted in nursing homes in Orange County, led by the University of California, Irvine.^19,20^ To gain a thorough understanding of these practices, the co-authors (SCW and VCCC) visited the University of California, Irvine, to observe the key practices. Patients received 10% povidone-iodine ointment in the anterior nares twice daily for 5 days (Monday through Friday) every other week. Chlorhexidine gluconate (CHG) (4% rinse-off antiseptic wash for assisted showering or 2% no-rinse cloths for bed bathing) was used twice weekly, irrespective of MRSA carriage. Frontline nursing staff and healthcare assistants were trained in the application of nasal povidone-iodine ointment, and the step-by-step procedure for CHG bathing, including preparing the CHG solution for use with bathing cloths.^19^

### Monitoring quality of MRSA decolonization

The adequacy of the povidone-iodine application was assessed using commercially available test strips (SenSafe®Iodine Test Strips, Industrial Test Systems, Inc., USA). During the application week, the ICN randomly selected 12 patients (6 male and 6 female) and swabbed their anterior nares two hours post-application. The ICN immersed the swab in 5 mL of sterile water, then dipped an iodine test strip into the sterile water for 10 seconds. The color of the test strip was then compared with a color chart to determine the iodine levels, ranging from 0 to 5 ppm. Mean iodine concentrations from nasal swabs were analyzed over the six-month study period to assess application consistency.

Each week, the ICN randomly selected 10 patients (5 male and 5 female) and scored the appropriateness of healthcare assistants’ bathing technique. Scoring included coverage of the face, neck, shoulders, and chest (1-point); abdomen (1-point); both arms and hands (1-point); anterior lower limbs (1-point); posterior lower limbs (1-point); back (1-point); buttocks (1-point); and groin (1-point). An additional point was awarded for each area if an adequate contact time of at least 1 minute was achieved, allowing for a maximum of 16 points for full compliance.

We measured residual chlorhexidine levels on patients’ skin after bathing using an in-house method, specificially quantifying the chlorhexidine component of CHG and expressing the levels as geometric mean and standard deviation (SD).^24^ The ICN randomly selected six patients (3 male and 3 female) twice weekly for sampling. The ICN swabbed a 5 cm × 5 cm area of skin on the anterior abdomen immediately after the patients were bathed and again at 8 and 24 hours. The ICN sent the swabs to the clinical microbiology laboratory, where the chlorhexidine levels were measured according to the laboratory protocol, as illustrated in the Supplementary File.

### Evaluation of MRSA decolonization

The ICN swabbed the nares, axilla, groin, and rectum of all patients in the infirmary unit at 2, 4, and 6 months after MRSA decolonization was initiated. The ICN immediately sent the swabs to the clinical microbiology laboratory for analysis. We monitored interval changes in the percent of MRSA nasal and skin colonization during the course of MRSA decolonization. We used the percent of gastrointestinal colonization as an internal control because we did not have a specific decolonization regimen for the gastrointestinal tract.

### Air and environmental cultures for MRSA

The ICN collected air samples in the infirmary unit using the settle plate method. We placed ChromID MRSA agar (bioMerieux, France) plates at 30 designated positions for an exposure duration of 2 hours once weekly (Figure 1). We collected environmental samples using flexible, premoistened sterile polywipe sponge swabs measuring 5 cm × 10 cm (Medical Wire and Equipment, UK), as previously described.^25,26^ Six designated sites were serially sampled once weekly, including two bathing trolleys and one sling in Ward 2B and Ward 2C of the infirmary unit. The ICN immediately sent the culture plates from the air samples and environmental sponge swabs to the clinical microbiology laboratory for analysis.

### Laboratory diagnosis of MRSA

Nasal, axillary, groin, and rectal swabs, as well as environmental sponge swabs, were processed with standard laboratory techniques using ChromID MRSA agar (bioMérieux, France) as previously described.^17,26,27^ Air samples collected with ChromID MRSA agar were directly incubated at 35–37°C for 24–48 hours under aerobic conditions. The environmental sponge swabs were squeezed repeatedly to ensure proper mixing in a sterile bag. Then, a 0.1 mL suspension from the bag was used for culture. Isolates that grew on the selective media were identified to the species level using matrix-assisted laser desorption/ionization time-of-flight mass spectrometry (MALDI-TOF MS) (Bruker Daltonics, Bremen, Germany). The susceptibility of the isolates was determined using the Kirby-Bauer disc diffusion method and was interpreted according to the Clinical and Laboratory Standards Institute breakpoints.

### Statistical analysis

The Student’s *t*-test was used to compare the participant characteristics. To assess changes in MRSA colonization over time among patients in the decolonization program, a generalized linear mixed model with a logit link was employed. This model accounted for the repeated measures at baseline, 2, 4, and 6 months. MRSA colonization status served as the dependent variable, while time points, age, and sex as fixed effects, while random effects addressed within-patient correlation across the time points. Odds ratios with 95% confidence intervals quantified the effects, with values <1 indicating a lower likelihood of MRSA. Linear regression was used to analyze the temporal trends of residual povidone-iodine concentrations in nasal swabs and MRSA contamination in air and environmental samples throughout the study. Statistical significance was set at *P* < .05.

## Results

### Epidemiology of MRSA colonization among patients in the infirmary unit

On the commencement date of the MRSA decolonization program, January 1, 2025, all 60 patients (35 female and 25 male) were recruited, with a mean age of 69 years and a SD of 15 years. The mean age of female patients was significantly higher than that of male patients (73 ± 15 years vs. 64 ± 13 years, *P* = .021). Among these 60 patients, the mean ± SD for length of stay before MRSA decolonization was 827 ± 459 days. Of these cases, 95% (57/60) were bedridden; 98% (59/60) were wearing diapers; 53% (32/60) had either a nasogastric tube or a percutaneous endoscopic gastrostomy tube feeding; 15% (9/60) had a tracheotomy, and 7% (4/60) had wounds.

At baseline, 47 of 60 patients (78%) carried MRSA. MRSA colonization varied significantly by body site. A significant difference in MRSA colonization rates was observed among the nasal (20/60, 33%), skin (39/60, 65%), and rectal sites (32/60, 53%), with a *P*-value of .002 indicating a statistically significant overall difference among these sites. Twelve patients (20%) had positive cultures from all three screening sites.

### Compliance with MRSA decolonization

**Figure 2.**
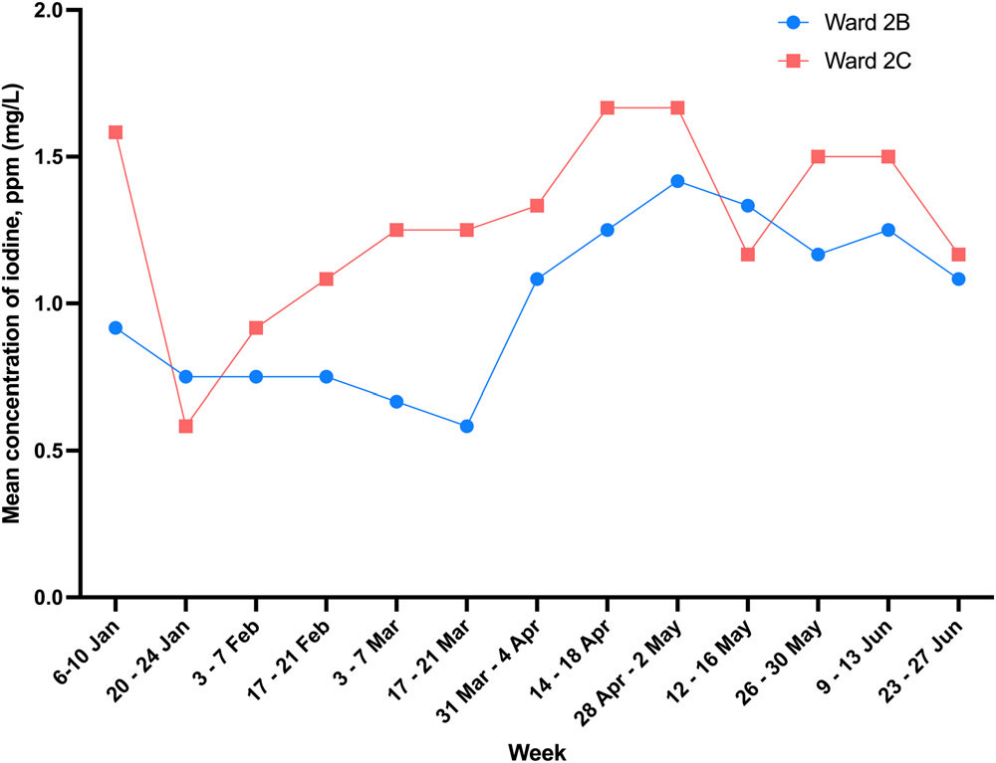
Iodine concentration in the anterior nares of patients in the Infirmary Unit, Grantham Hospital.

We collected 156 nasal swabs during the 13 sampling weeks of our six-month intervention period to measure residual povidone-iodine concentrations, which served as a proxy for staff compliance. The mean ± SD concentration of iodine was 1.1 ± 0.7 ppm (mg/L) (Figure 2). The iodine concentration in Ward 2C did not change significantly over time (slope = 0.033; *R* ^2^ = 0.034; *P* = .105), indicating that compliance was stable and consistently maintained throughout the study. In contrast, we found a statistically significant positive trend (slope = 0.050; *R* ^2^ = 0.089; *P* = .008) in Ward 2B. This result indicated that the consistency and/or amount of povidone-iodine application gradually improved over the six-month period, suggesting that the efforts to encourage staff compliance with the protocol were successful.

**Figure 3.**
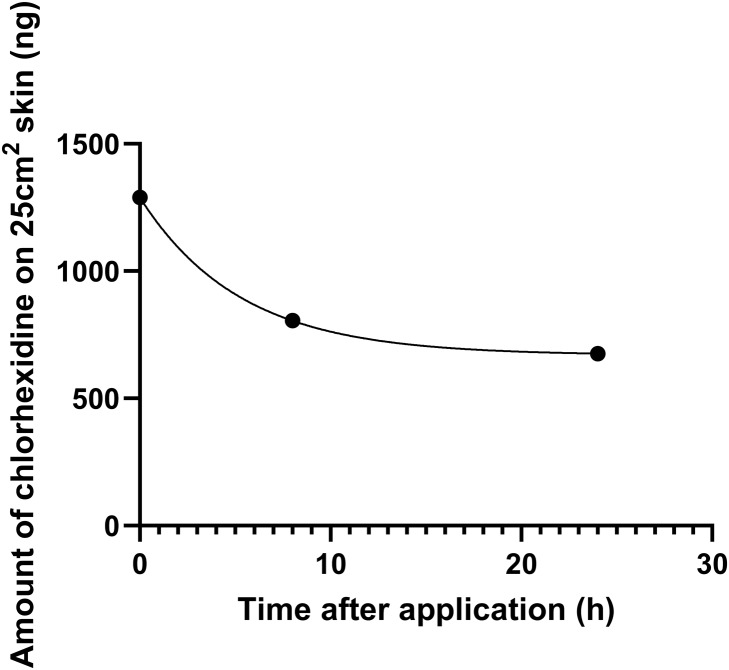
Geometric mean of chlorhexidine content on the skin of patients in the Infirmary Unit, Grantham Hospital. *Note:* Of the 30 patients randomly selected for measurement of chlorhexidine levels on the skin of the anterior abdomen immediately after bathing, as well as at 8 hours and 24 hours thereafter, the geometric mean and standard deviation of chlorhexidine levels were 1290 ± 3 ng, 805 ± 3 ng, and 676 ± 2 ng, respectively.

During the first 2 months, 97.2% (243/250) of the bathing observations were fully compliant with the bathing technique. Throughout the entire study period, staff were awarded 99.7% (3,986/4,000) of the possible points for the bathing technique. The chlorhexidine levels (geometric mean and SD) on the anterior abdominal skin, immediately after bathing and at 8 hours and 24 hours thereafter, were 1290 ± 3 ng, 805 ± 3 ng, and 676 ± 2 ng, respectively (Figure 3).

### Evaluation of MRSA decolonization

The MRSA colonization rate at any body site decreased significantly among the 60 patients in the MRSA decolonization cohort during the six months after the initiation of intervention (*P* = .040). MRSA colonization of the skin decreased significantly from 65% to 29% (*P* < .001), but MRSA colonization of the nares (*P* = .557) and rectum (*P* = .261) did not (Table 1).

**Table 1.** Analysis of change in MRSA colonization rate among patients in the Infirmary unit, Grantham Hospital using generalized linear mixed model

	MRSA colonization rate	Odd ratio (95% confident interval)^a^	*P*-value
MRSA (any site)			
Time			.040^b^
Baseline (reference)	47/60 (78%)	NA	NA
2 months	43/58 (74%)	0.768 (0.383–1.538)	.453
4 months	34/53 (64%)	0.450 (0.225–0.904)	.025
6 months	32/51 (63%)	0.402 (0.195–0.826)	.014
MRSA (nasal or skin)			
Time			<.001^b^
Baseline (reference)	46/60 (77%)	NA	NA
2 months	42/58 (72%)	0.780 (0.385–1.583)	.489
4 months	26/53 (49%)	0.260 (0.126–0.533)	<.001
6 months	20/51 (39%)	0.163 (0.076–0.351)	<.001
MRSA (nasal)			
Time			.557^b^
Baseline (reference)	20/60 (33%)	NA	NA
2 months	17/56 (30%)^c^	0.894 (0.488–1.639)	.715
4 months	16/53 (30%)	0.895 (0.466–1.721)	.738
6 months	12/51 (24%)	0.604 (0.290–1.257)	.175
MRSA (skin)			
Time			<.001^b^
Baseline (reference)	39/60 (65%)	NA	NA
2 months	37/58 (64%)	0.913 (0.509–1.640)	.760
4 months	20/53 (38%)	0.280 (0.144–0.546)	<.001
6 months	15/51 (29%)	0.182 (0.085–0.387)	<.001
MRSA (rectal)			
Time			.261^b^
Baseline (reference)	32/60 (53%)	NA	NA
2 months	24/58 (41%)	0.576 (0.323–1.029)	.062
4 months	21/53 (40%)	0.536 (0.251–1.143)	.105
6 months	23/51 (45%)	0.707 (0.306–1.578)	.394

a Based on the model adjusted for age and sex of patients.

b *P*-value of the difference across all time points.

c Two patients refused to have nasal swabs collected for MRSA screening.

### Air and environmental cultures for MRSA

**Figure 4.**
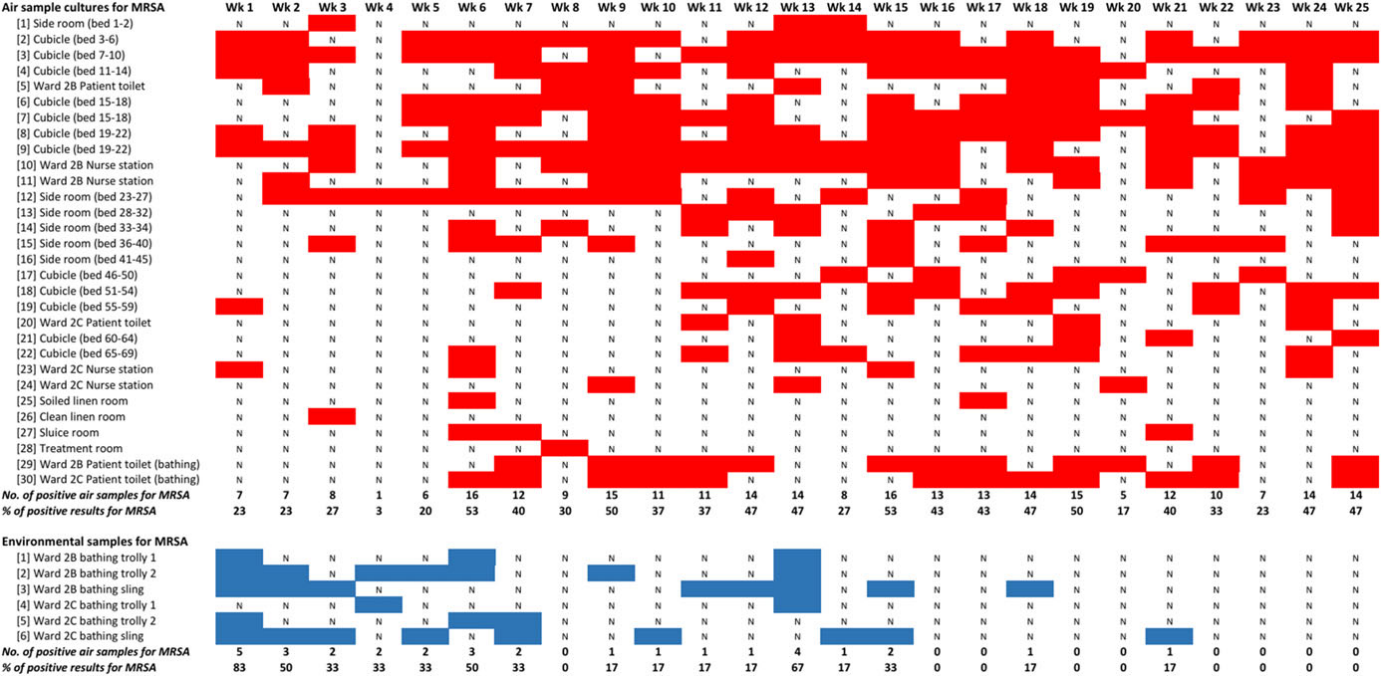
Weekly MRSA culture results from air and environmental samples in relation to the collection sites in the Infirmary unit, Grantham Hospital. *Note:* Bathing trolley, a bathing trolley is a wheeled cart designed to assist with patient bathing and personal hygiene. It typically includes storage for bathing supplies such as chlorhexidine gluconate, towels, and other personal care items, making it easier for healthcare staff to provide bathing assistance to patients, particularly those with limited mobility. The red bar represents MRSA-positive culture in the air samples, while the blue bar represents MRSA-positive culture in the environmental samples. “N” represents MRSA-negative cultures for either air or environmental samples.

**Figure 5.**
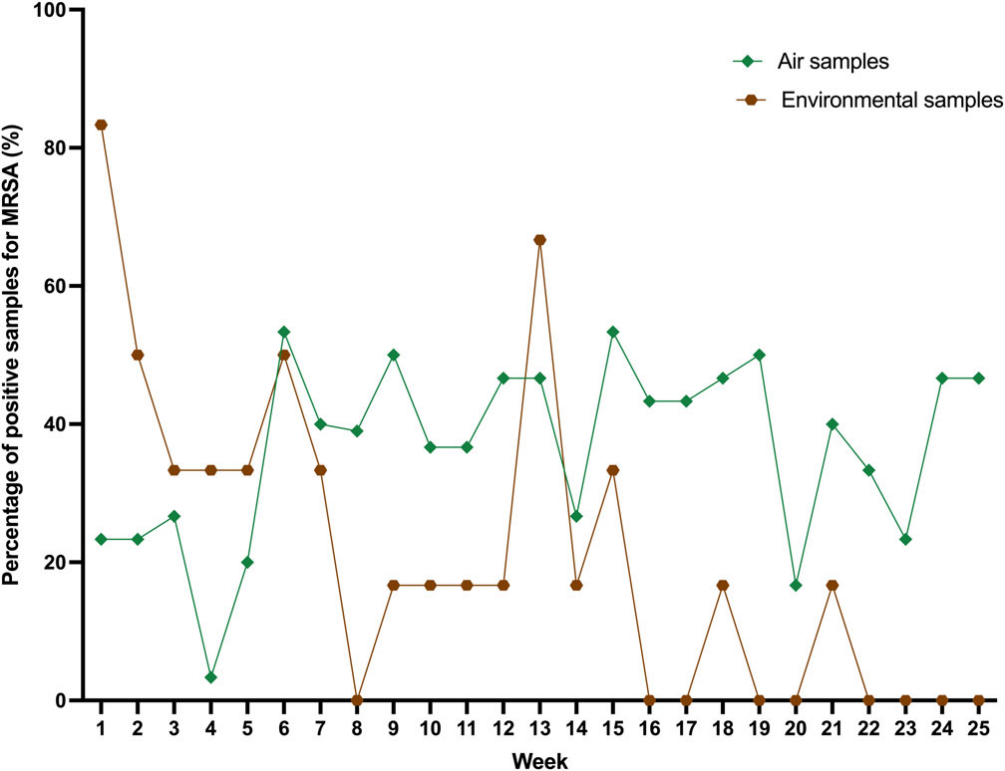
Weekly percentage of MRSA-positive cultures in the Infirmary unit, Grantham Hospital.

During the six-month intervention period (January to June 2025), we collected 750 air samples and 150 environmental samples over 25 weeks. Overall, 272 of 750 air samples (36%) and 32 of 150 environmental samples (21%) grew MRSA (Figure 4). The percentage of MRSA-positive air samples did not change significantly over time (slope = 0.601; *R* ^2^ = 0.116; *P* = .096) (Figure 5). In contrast, the percentage of MRSA-positive environmental samples from bathing trolleys and slings decreased significantly (slope = −2.205; *R* ^2^ = 0.505; *P* < .001).

## Discussion

We successfully demonstrated universal MRSA decolonization in the long-term care setting, marking the first successful implementation in Hong Kong. Given the increasing burden of MRSA in our RCHEs,^12–18^ our previous attempt at universal MRSA decolonization in these facilities resulted only in a reduction in hospital admissions due to MRSA infections, without a corresponding decrease in colonization within the RCHEs.^28^ Suboptimal compliance among healthcare workers remained a critical issue. To address this challenge, we adapted the Orange County MRSA decolonization protocol, placing particular emphasis on meticulous training and compliance monitoring.^19^ As a result, the infirmary unit has achieved a significant reduction in MRSA colonization and environmental contamination over a period of six months.

The baseline overall MRSA colonization prevalence (i.e., a positive culture of the nares, skin, or rectum) in the infirmary unit was 78%, which is 30% higher than that of RCHEs within our healthcare network.^17^ This high level of MRSA colonization is not unexpected, as over 90% of patients were bedridden and required frequent nursing care procedures.

MRSA colonization decreased over the six-month intervention period, driven primarily by a 55% reduction in MRSA skin colonization. Gastrointestinal colonization did not change significantly. MRSA nasal colonization decreased by one-third, but this was not statistically significant, possibly due to the small sample size of 51 subjects at the end of follow-up. Our finding that MRSA nasal colonization did not significantly decrease despite good compliance with povidone-iodine application and measurable residual concentrations may also be explained by the inferiority of nasal iodophor compared with mupirocin, as reported in a recent study.^29^ We did not initiate oral antimicrobials, such as trimethoprim/sulfamethoxazole plus rifampin, to reduce gastrointestinal MRSA colonization; consequently, the prevalence of gastrointestinal MRSA colonization did not decrease. Thus, persistent MRSA colonization of the gastrointestinal tract may have reduced the effectiveness of our intervention. Nevertheless, prior trials have shown success reducing serious infections, hospitalization, and multidrug-resistant organisms through topical decolonization alone.^19,20,29^

A significant reduction in the skin MRSA colonization rate was observed at the four-month and six-month time points, but not at two months after the initiation of this program. This is likely attributed to suboptimal CHG bathing technique during the first two months. Consistent with ongoing MRSA shed from suboptimally decolonized patients, weekly environmental samples from bathing trolleys and slings collected in patients’ bathrooms also had a higher MRSA contamination rate during this time. In response to the interim MRSA screening results at two months, we conducted intensive staff training and enforced environmental disinfection, especially of the bathing facilities. Subsequently, the bathing technique became fully compliant, as indicated by our audit, and we observed statistically significant decreasing trend in MRSA colonization and environmental positivity from bathing trolleys and slings. These findings demonstrate that data collected from close monitoring of practices, together with interval MRSA screening, can provide insights for infection prevention programs to develop timely feedback and education for staff, ultimately improving outcomes.

We used the settle plate method to collect air samples weekly from both clinical and non-clinical areas in the two study wards as a novel way to monitor the effect of our MRSA decolonization program. Approximately one-third of the air samples tested positive for MRSA, and the percentage of positive cultures did not decrease during the study period. This finding suggests that air dispersal of MRSA was not correspondingly reduced, despite a substantial decrease in MRSA skin and nasal colonization. The infirmary unit was equipped with window air conditioners and portable air purifiers to maintain indoor air ventilation,^23^ which was similar to the ventilation systems in RCHEs, where we previously demonstrated the dispersal of MRSA through the air.^17^ If we had left the settle plates in place longer (e.g., 24 hours instead of 2 hours), we may have been better able to detect changes in the MRSA burden in the air. In addition, MRSA dispersal is strongly related to patient care activities.^31^ We placed the settle plates in the wards during morning diaper changes. There appears to be a clustering effect for air sample culture positivity in cubicles with bed numbers 1–22, located in Ward 2B, which is a male ward. Further studies are warranted to assess the correlation between patients’ MRSA colonization, including gender status, and MRSA positivity in the air during decolonization programs across different clinical settings.

We monitored chlorhexidine levels on the skin among randomly selected patients undergoing decolonization immediately after bathing, as well as at eight hours and 24 hours after bathing. Since the measurement of chlorhexidine skin concentration is rarely performed and lacks a standard method in the literature, particularly regarding the timing of sampling and the use of dry versus wet swabs,^32,33^ we adopted a recently published protocol with modifications using ultra-high-performance liquid chromatography-tandem mass spectrometry.^24^ Our findings indicated that all randomly selected patients had detectable chlorhexidine at 24 hours after bathing. The persistence of chlorhexidine on the skin can facilitate the clearance of MRSA.^34^ Further studies are required to investigate measurement standardization and the lasting effects of chlorhexidine on the skin. This information could help us better estimate the optimal interval between baths, especially since bathing may be not be done frequently in infirmary units or RCHEs.

This study has several limitations. First, our findings in the infirmary setting may not be generalizable to RCHEs. However, the stable patient population in this infirmary facilitated the interpretation of the results of our decolonization program. In contrast, residents of RCHEs are frequently admitted to hospitals and have a higher risk of MRSA acquisition, which complicates the assessment of the efficacy of intervention. Second, our protocol did not reduce MRSA in the air. We hypothesize that this failure was related to persistent carriers who continued to shed the organism.^35^ Therefore, facilities implementing this protocol should be aware that routine patient care activities can facilitate MRSA dispersion from patients who persistently carry MRSA.^31,36^ Third, the six-month study period was relatively short. However, we are continuing to monitor MRSA colonization while ensuring high-quality CHG bathing along with infection control measures to prevent MRSA acquisition among newly admitted patients. As gastrointestinal colonization of MRSA is gradually lost over time, MRSA colonization may continue to wane over time in this population whose median survival is estimated to be 2.4 years.^27^

## Conclusion

This study successfully implemented a universal MRSA decolonization program in a long-term care setting in Hong Kong, achieving significant reductions in overall MRSA colonization, particularly at skin sites, along with decreases in MRSA environmental contamination. Although gastrointestinal colonization and air dispersal were still observed, our findings indicate that long-term care facilities could reduce MRSA prevalence by adopting universal decolonization practices and by providing comprehensive training.

## Supporting information

Wong et al. supplementary material Wong et al. supplementary material

## Data Availability

The datasets generated for this study will be made available in anonymized form from the corresponding author upon reasonable request.

## Supplementary material

The supplementary material for this article can be found at https://doi.org/10.1017/ice.2025.10388.
