# Supplementary material for: Bridging borders: adapting the Orange County methicillin-resistant Staphylococcus aureus decolonization protocol for an infirmary unit in Hong Kong
Source: Infect Control Hosp Epidemiol. 2026 Jan 23;47(4):357–64. doi: 10.1017/ice.2025.10388 (PMC13216797; doi:10.1017/ice.2025.10388)
Supplement: Wong et al. supplementary material [file S0899823X25103887sup001.docx]

Supplementary File

Laboratory protocol to measure chlorhexidine levels

The swab is immersed in 2 mL of methanol for extraction. The extraction of chlorhexidine begins by transferring all the methanol to a new tube, which is then dried using a speed-vac concentrator. The swab is transferred to a new tube containing 0.5mL of extraction buffer (50% v/v methanol, 10mM Ammonium Formate, 0.1% Formic Acid). The tube is vortexed for 1 min and incubated in a water bath sonicator for 20 min at 100W. The liquid is then transferred back to the previous tube in the speed-vac. This sonication process is repeated with another 0.5 mL of extraction buffer. All extraction buffer is combined into the previous tube until it is completely dry, and the tubes are stored at -20^o^C until mass spectrometry analysis. The tubes are reconstituted in 1 mL of extraction buffer, followed by 1 min of vortexing, 5 min of sonication, and 10 min of centrifugation at 10,000rpm. Finally, the extraction buffer is being transferred to a new glass vial for analysis by liquid chromatography–mass spectrometry (LC-MS).

Quantification of chlorhexidine was performed using ultra-performance liquid chromatography coupled with a triple quadrupole linear ion trap mass spectrometer (UPLC-Q-TRAP 5500) analytical platform (Applied Biosystems Instrument Corporation, Foster city, CA). The instrument was operated in positive ionization mode with multiple reaction monitoring (MRM) acquisition. Chromatography was performed on a Waters ACQUITY BEH C18 column (100 × 2.1 mm; 1.7 µm, Waters; Milford, MA, USA). The column and autosampler temperatures were maintained at 40°C and 4°C, respectively. The injection volume was 3 µL. The settings for optimal signals for the ions pairs of chlorhexidine followed the previous publication [1]. Helium was chosen as the collision gas. Linearity of the calibration curve was determined for chlorhexidine concentrations of 39.063, 78.125, 156.25, 312.5, 625, 1250, 2500, 5000 ng/mL. The quantity of the chlorhexidine in the eluate was determined by Analyst (version 1.6.3).

Reference

1. Zhao X, Schaffzin JK, Carson J, *et al*. Analysis of chlorhexidine gluconate in skin using tape stripping and ultrahigh-performance liquid chromatography-tandem mass spectrometry. *J Pharm Biomed Anal* 2020;183:113111.
